# Case Study – Restarting Clozapine in a Patient With Treatment Resistant Schizophrenia

**DOI:** 10.1192/bjo.2025.10771

**Published:** 2025-06-20

**Authors:** Sabaritha Subramania Pillai, Chris Sage

**Affiliations:** Kent and Medway NHS and Social Care Partnership Trust, Kent, United Kingdom

## Abstract

**Aims:** Clozapine is the most effective treatment in patients with treatment resistant Schizophrenia as recommended by the National Institute of Health and Care Excellence (NICE). This case study aims to explore a patient’s journey in mental health services during the relapse of his mental illness and the significance of reconsidering clozapine in patients who responded well to the medicine before.

**Methods:** 35-year-old male diagnosed with Paranoid Schizophrenia admitted to the acute ward following relapse of his mental illness. He had many admissions under sections in the past and was tried on several antipsychotics. Historically he has shown non-compliance with his medications and poor engagement with the mental health services. In the current presentation, he was found to be floridly psychotic with paranoid and persecutory delusions. He mentioned that people were coming to his flat through the walls and injected him with drugs. He was prescribed clozapine in his last admission as he met the criteria for treatment resistant Schizophrenia. During his stay, we tried him on a combination of non-clozapine antipsychotics due to the history of poor drug compliance. Since he didn’t show any response after trying other antipsychotics for a longer period of time, the team decided to restart him with clozapine. Patient showed a good response following this.

**Results:** In the above case scenario, though the patient previously responded well to clozapine, the team was reluctant to restart clozapine because of the following reasons: history of poor drug concordance, sudden withdrawal of clozapine leads to various side effects like withdrawal associated psychosis and catatonia, cholinergic rebound and rarely it causes serotonergic symptoms. The pharmacological profile of clozapine is complex as it induces a range of neuronal changes at the receptors particularly when taken for a prolonged period of time. Evidence suggests that these neuronal changes are responsible for the occurrence of withdrawal symptoms on clozapine discontinuation.

**Conclusion:** Evidence suggests that one third of patients with schizophrenia are treatment resistant and clozapine is the treatment of choice for refractory psychosis. Although the literature says that sudden stoppage of clozapine is associated with an array of withdrawal symptoms, it is important to consider restarting the patient with clozapine, especially if they showed a good response in the past. This case study also highlights the good practice for communicating with patients about the significance of clozapine, its effect and withdrawal symptoms due to discontinuation which helps treatment adherence.

